# 164. Restriction of Antimicrobials Dispensing without Prescription on a National Level: Impact on the Overall Antimicrobial Utilization in Community Pharmacies in Saudi Arabia

**DOI:** 10.1093/ofid/ofab466.366

**Published:** 2021-12-04

**Authors:** Khalid Eljaaly, Ahmed Al-Jedai, Yasser Almogbel, Nasser Alqahtani, Hajer Almudaiheem, Nancy Awad, Dema Alissa, Abdullah Assiri, Tareef Alaama

**Affiliations:** 1 King Abdulaziz University, Jeddah, Makkah, Saudi Arabia; 2 Ministry of Health, Riyadh, Ar Riyad, Saudi Arabia; 3 Qassim University, Buraidah, Al Qasim, Saudi Arabia; 4 Riyadh first health cluster, Riyadh, Ar Riyad, Saudi Arabia; 5 IQVIA, Dubai, Dubai, United Arab Emirates

## Abstract

**Background:**

High rates of non-prescription dispending of antimicrobials has led to a significant increase in antimicrobial overuse and misuse in Saudi Arabia (SA). The objective of this study was to evaluate antimicrobial utilization following enforcement of a new prescription-only antimicrobial dispensing policy in the community pharmacy setting in SA.

**Methods:**

Data were extracted from the IQVIA database between May 2017 and May 2019. Antimicrobial consumption rate based on the sales, defined daily dose in grams (DDD), DDD/1000 inhabitants’/day (DID), and antimicrobial claims for pre-policy (May 2017 to April 2018) and post-policy (June 2018 to May 2019) periods was assessed.

**Results:**

Overall antimicrobial utilization slightly declined (~9-10%) in post-policy vs. pre-policy period (sales, 31,334 vs.34,492 thousand units; DDD, 183,134 vs. 202,936 thousand grams), with an increase in the number of claims (~16%) after policy implementation. There was a sudden drop in the consumption rate immediately after policy enforcement; however, the values increased subsequently, matching closely to the pre-policy values. Consumption patterns were similar in both periods. Penicillins were the most commonly used antimicrobial (sales, 14,700 - 11,648 thousand units; DDD, 71,038 - 91,227 thousand grams; DID, 2.88 - 3.78). For both the periods, the highest dip in utilization was observed in July (sales, 1,027 - 1,559 thousand units; DDD, 6,194 - 9,399 thousand grams), while the highest spike was in March/October (sales, 3,346 - 3,884 thousand units; DDD, 22,329 - 19,453 thousand grams).

**Conclusion:**

Non-prescription antimicrobial utilization reduced minimally following policy implementation in the community pharmacy setting across SA. Measures to aid effective implementation of prescription-only regulations are necessary.

**Disclosures:**

**All Authors**: No reported disclosures

